# Self-reported adherence with the use of a device in a clinical trial as validated by electronic monitors: the VIBES study

**DOI:** 10.1186/1471-2288-12-171

**Published:** 2012-11-14

**Authors:** Brianne A Jeffrey, Marian T Hannan, Emily K Quinn, Sheryl Zimmerman, Bruce A Barton, Clinton T Rubin, Douglas P Kiel

**Affiliations:** 1 Hebrew SeniorLife Institute for Aging Research, Musculoskeletal Research Center, Boston, MA, USA; 2 Harvard Medical School, Department of Medicine, Boston, MA, USA; 3 Boston University School of Public Health, Data Coordinating Center, Boston, MA, USA; 4 Cecil G. Sheps Center for Health Services Research Chapel Hill, The University of North Carolina, Chapel Hill, NC, 27412, USA; 5 University of Massachusetts Medical School, Worcester, MA, 01655, USA; 6 Department of Biomedical Engineering Stony Brook, SUNY Stony Brook, New York, NY, 10027, USA

## Abstract

**Background:**

Adherences to treatments that require a behavioral action often rely on self-reported recall, yet it is vital to determine whether real time self reporting of adherence using a simple logbook accurately captures adherence. The purpose of this study was to determine whether real time self-reported adherence is an accurate measurement of device usage during a clinical trial by comparing it to electronic recording.

**Methods:**

Using data collected from older adult men and women (N=135, mean age 82.3 yrs; range 66 to 98 yrs) participating in a clinical trial evaluating a vibrating platform for the treatment of osteoporosis, daily adherence to platform treatment was monitored using both self-reported written logs and electronically recorded radio-frequency identification card usage, enabling a direct comparison of the two methods over one year. Agreement between methods was also evaluated after stratification by age, gender, time in study, and cognition status.

**Results:**

The two methods were in high agreement (overall intraclass correlation coefficient = 0.96). The agreement between the two methods did not differ between age groups, sex, time in study and cognitive function.

**Conclusions:**

Using a log book to report adherence to a daily intervention requiring a behavioral action in older adults is an accurate and simple approach to use in clinical trials, as evidenced by the high degree of concordance with an electronic monitor.

**Trial registration:**

Clinicaltrials.gov NCT00396994

## Background

Adherence to treatment, whether in clinical trials or clinical practice, is important to monitor because the full benefit of a treatment regimen may only be understood and achieved if the regime is followed [1-5]. Poor treatment adherence for approved medical interventions can lead to exacerbation of disease and other declining health conditions, avoidable hospitalizations, drug- resistance, and higher costs for both patients and providers [2,3,5-10]. In fact, the U.S. costs of managing poor adherence have been estimated to be as much as $100 billion annually [2,3,6]. In clinical trials, poor adherence makes it difficult to draw conclusions about the implications of a trial or treatment, as results may be dose-dependent or rely heavily on a strict adherence to a prescribed regimen [1,3-5,8]. Without knowing the optimal way to monitor adherence, one cannot accurately interpret the results of clinical trials or properly treat patients [1,7].

Adherence to treatment is monitored by various methods including self-report, proxy report, observation, and in the case of medication use, by pill counts and more recently by Medication Event Monitoring Systems (MEMS) pill bottle caps [1,2,7]. MEMS caps serve as a form of electronic monitoring by recording when the cap is opened or pill removed, and have emerged as a preferred, objective standard of adherence monitoring in many clinical trials. [1]. Similar to these studies focusing on medication use, electronic monitoring of device adherence could be considered as a gold standard to monitor adherence.

In clinical trials, which often rely on self-reported recall of adherence, levels of adherence tend to be higher than what is observed in actual practice once a treatment is approved. [1] Thus there is even more of a need to be able to monitor adherence when a new treatment is prescribed. Often clinical trials requiring that participants perform a daily action such as exercise, rely on self-reporting of adherence using approaches such as diaries or log books that are signed each day that an individual adheres to the treatment. The accuracy of such self-reporting in trials has not been investigated because there is rarely a method of confirming the accuracy of the log book. The use of electronic adherence monitoring may provide such a measure.

A range of behavioral treatments and devices have become part of the care of older adults with a variety of chronic diseases (e.g. diet and exercise plans, blood glucose testing devices, TENS units). As the use of treatments involving a behavioral intervention become more common, monitoring adherence of their implementation will be as important as monitoring drug adherence. To date, adherence to treatment with devices has not been well-studied, particularly in older adults. Therefore the purpose of this study was to determine the accuracy of a simple real time reporting of adherence to a behavioral treatment in a clinical trial with a daily log book by comparing it to the gold standard electronic recording via the use of a radio frequency identification card reader. The device used in this trial was one that delivered high frequency, low magnitude, mechanical stimulation to improve bone mass, and it was delivered in a randomized, sham platform-controlled trial, as previously described.[11]

## Methods

All aspects of the study were reviewed and approved by the Institutional Review Board at Hebrew Rehabilitation Center. The “VIBES” (Vibration to Improve Bone Density in Elderly Subjects) study is a randomized, double-blind, sham platform-controlled device trial of the efficacy of daily low magnitude, high frequency vibration to increase bone mineral density, balance, muscle mass and strength in men and women over the age of 60 who reside in independent living settings, and who have osteopenia of the hip or spine (between −1 and −2.5 standard deviations below young normal reference values) at baseline testing. The sample and the inclusion and exclusion criteria have been described previously [11], but in general, participants had an average age of 82.3 years (range 65–103) and were generally cognitively intact, relatively well-educated, mostly Caucasian men and women living around the Boston area. Study participants were recruited from 15 independent living facilities and informed consent was obtained from all.

VIBES participants were asked to stand on a vibrating platform seven days a week, for ten minutes each day, for up to three years. The device delivered a vibration at 30Hz (cycles per second), 0.3g acceleration (where g is earth’s gravitational field, or 9.8 meters per second squared). This low magnitude is often described as a buzzing sensation, and is considered safe by ISO standards for up to 4 hours each day. The sham group stood on an identical device that did not deliver this same vibration. There was no scheduled time for treatment on any given day; participants were simply instructed to use the device once a day at a time convenient to them. Daily adherence to study treatment was tracked using both electronic as well as self-reported written logs. More specifically, each day when participants used the vibrating platforms, they were asked to indicate the date and time of their session in a paper log book. Their session time on the VIBES platform was also recorded by the machine itself using unique person-specific radiofrequency identification (RFID) card system.

### Self-reported paper logs

Participants were asked to sign a pre-designed paper log book at the start of each daily session; the log book was located in the same room as the platform. Dates were listed in a column with a row of empty boxes for participants to initial under their name. On the first study day of platform use, participants were individually instructed by research staff to write their initials under their name on the proper date and to note the approximate time that they stepped onto the platform. Paper logs were collected bimonthly and manually double entered into a database over the first year of the clinical trial.

### Electronic monitoring

Each platform session was initiated by an assigned RFID card specific to the person and his or her assigned platform. Sham platforms used in this trial were initiated by a similar card but participants assigned to these platforms did not receive the prescribed low magnitude vibration. Each participant was given his or her own card to use and instructed to use only that RFID card. When the machine was activated, a record was created in the platform computer memory card containing the date and time, length of the session, and any interruption during the 10-minute session. Each platform’s memory device was periodically downloaded and sent to the data coordinating center.

Early in the study, while reviewing the RFID recorded data from several platforms, we observed the occurrence of multiple sessions by individuals on the same date; further, they were recorded as taking place at late hours during the night. Ultimately it was discovered that the odd hours and duplicate sessions could be accounted for by a platform clock malfunction in 10 of the 38 platforms at 4 of the 16 study sites and that a simple subtraction of 12 hours corrected the problem. It should be noted that paper logs were used to confirm the 12-hour clock error. Two additional platforms reported similar problems but a 12 hour correction did not appear to be the explanation; instead, comparison with the paper logs indicated a discrepancy such that one clock time was ahead by 4.5 hours and the other by 5 hours. A total of 44 out of 136 participants were affected by these adjustments. These errors were corrected and the corrected data for these observations were retained in the analyses, as we felt the corrected clock errors would not invalidate our ability to compare the two methods.

In a few additional cases we discovered that duplicate sessions recorded by the devices resulted from RFID card sharing between participants or due to a participant attempting to do two sessions in one day after missing the previous day. Unlike the correction of the clock errors before analysis (noted above), these observations were not corrected before analysis because they were felt to be due to the behavior of the individual participants.

### Participant characteristics

Age was obtained by interview, and height and weight were measured using a calibrated scale and stadiometer at baseline. Cognitive status was assessed using the Short Blessed Test (SBT) [12] during the consenting process. Time in study was calculated as the number of days from the first time that a participant logged in or initiated an electronically recorded session until the last day the same participant recorded a session by either method for up to one year of participation. In some instances the last day occurred because the participant dropped out (N=2), or because an individual participant had accumulated less than a year of follow up when the data were locked for analysis (N=56). a A health problems list was used to query whether a participant had been told by a physician in the past year that he/she has the listed disease or chronic health problem.

### Analysis

Adherence data were included from participants who had been in the study between 6 and 12 months. Adherence for both the self-reported paper logs and electronically monitored methods was calculated by dividing the number of completed sessions by the number of sessions expected as per protocol. The protocol specified that 10 minute sessions should occur daily; however, since the paper logs did not include the length of treatment, adherence recorded by the devices was defined as the number of sessions initiated relative to the number of possible sessions.

We used an intraclass correlation coefficient (ICC) to determine agreement between the two methods of adherence measurement. We used an ICC, rather than the standard paired intraclass correlation, because the data were organized by adherence method, rather than paired measures by individual, and because we were primarily interested in the similarity of the measured adherence between methods. ICC was calculated via two-way mixed effects modelling, treating subject as a random effect and method of adherence as a fixed effect with a consistency definition as described by McGraw and Wong. [13]

A priori we hypothesized that the agreement between self-reported and electronically monitored adherence recording might differ by sex, age, number of months in the study, and cognition. Therefore, we stratified the ICC analyses by age (less than versus greater than or equal to the median), sex, cognition, based on Short Blessed Test scores (less than versus greater than or equal to the median), and time in study. Expecting that adherence might change over time, we divided the time in study into several groups; the minimum required six months participation time to less than nine months, nine months to less than twelve months, and twelve months. We used SAS Version 9.2 (SAS Institute, Cary, NC) for all analyses.

To examine individual level data, we plotted electronic adherence versus self reported adherence using a Bland-Altman plot. This plot provided an indication as to whether the difference (discrepancy) between the two measures of adherence was related to the level of adherence. (i.e., there is a systematic bias in the measurements) or the difference was randomly distributed across the range of adherence (i.e., measurements are unbiased). Individual observations outside of the two standard deviation limits are indicative of particularly large discrepancies.

## Results

Table 1 provides a summary of participant characteristics included in the analyses. The study sample was two thirds women and overwhelmingly Caucasian (n=130, 96.3%). Average age was 82.3 (SD 7.1), with 45% of participants ages 84 years or older. Not unexpectedly, since individuals with impaired cognitive status (Short Blessed Test >12) were excluded, the Short Blessed scores were relatively low (mean 2.4, SD 2.7). One quarter of the sample had 6 but less than 9 months of data, 19% had 9 but less than 12 months of follow up, and 56% of the sample had 12 months of follow up. On average, participants had slightly more than 3 health problems.

**Table 1:** Baseline characteristics of 135 participants included in the VIBES study

**Characteristic**	**N (%) or mean (SD)**
Percent female	90 (67%)
Age Group	
Age < 84	74 (55%)
Age ≥ 84	61 (45%)
Age (yrs)	82.3 (7.1)
Weight, lbs	157.6 (27.7)
Height, in	63.9 (4.0)
Short Blessed Test Score	2.4 (2.7)
Time in Study	
6 - <9 Months	34 (25%)
9 - <12 Months	25 (19%)
12 Months	76 (56%)
Time in Study (Months)	10.2 (2.4)
Education	
Not a College Graduate	64 (47%)
College Graduate	71 (53%)
Marital Status	
Never married/Divorced	24 (18%)
Widow or Widower	65 (48%)
Married	46 (34%)
Number of health problems	3.4 (2.1)

Two participants were excluded from our study comparison due to personal characteristics that interfered with the study goals. One participant was legally blind and unable to sign into the paper log sheet, while the other experienced rapid cognitive decline which compromised completing the daily attendance log book.

Results for self-reported and electronically monitored adherence are provided in Table 2. Overall, average electronic adherence was shown to be 0.76± 0.23 and self-reported adherence was 0.78± 0.23. Across all subgroups, self-reported adherence was greater than electronic adherence although these differences were small and not statistically different. For example, the greatest difference was observed in participants aged 84 and older, where the mean self-reported adherence was .77± 0.26 while the electronic log reflected a mean of 0.74±0.26.

**Table 2:** **Comparison of electronic versus self**-**reported adherence by age group**, **sex**, **time in study and cognition**

			**Electronic Adherence**		**Self**-**Reported Adherence**	
**Subgroup**		**Possible Days**	Days Recorded*	**Mean** (± **SD**)	**Days Reported**^†^	**Mean** (± **SD**)
Overall		42,098	31,902	0.76 (± 0.23)	32,678	0.78 (± 0.23)
Age Group	Age < 84	22,001	17,271	0.78 (±0.20)	17,496	0.79 (± 0.19)
	Age ≥ 84	20,097	14,631	0.74 (±0.26)	15,182	0.77 (± 0.26)
Gender	Male	14,680	10,969	0.76 (±0.25)	11,247	0.78 (± 0.26)
	Female	27,418	20,933	0.76 (±0.22)	21,431	0.78 (± 0.21)
Time in Study	6 - <9 Months	6,830	5,378	0.79 (±0.22)	5,440	0.80 (± 0.22)
	9 - <12 Months	7,452	5,358	0.73 (±0.27)	5,622	0.76 (± 0.26)
	12 Months	27,816	21,166	0.76 (±0.22)	21,616	0.78 (± 0.22)
Short Blessed Test Score	< 2 (Median)	17,889	14,001	0.78 (±0.20)	14,287	0.80 (± 0.19)
	≥ 2 (Median)	24,209	17,901	0.75 (±0.25)	18,391	0.77 (± 0.25)

^*^refers to the actual number of days recorded using the electronic monitor.

^†^refers to actual number of days recorded as being adherent on the self-reported log book.

**Figure 1:**
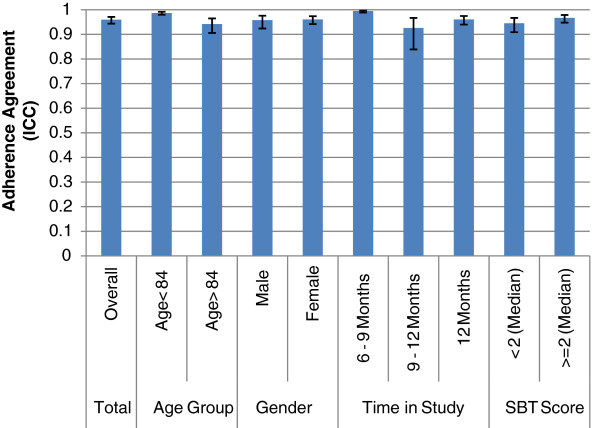
**Intraclass correlation with 95% confidence intervals of agreement between the two methods of adherence according to age, sex, time in study, and cognition.** SBT refers to the Short Blessed Test.

Agreement between self reported adherence and electronically monitored adherence is shown graphically in Figure 1. In general, we found the two methods to be in agreement, with an overall intra-class correlation of 0.96. The same high correlations were observed within different age groups, in men and women, time in study, and in the two categories of scores on the Short Blessed Test. Subgroup intra-class correlations ranged from 0.93 to 1.00.

**Figure 2:**
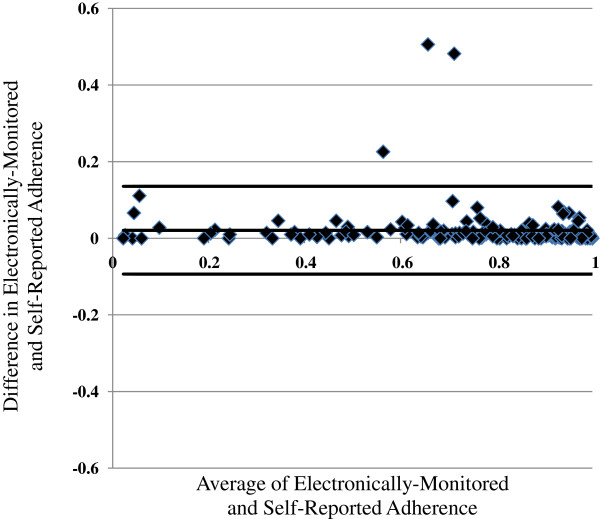
**Bland**-**Altman plot of the difference between electronically monitored adherence and self-reported adherence as compared with the mean of the two adherence measures.** The thick black lines indicate 2 SDs above and below the difference in adherence.

Examining individual level data, the Bland-Altman plot (Figure 2) in Figure 2 again displays excellent agreement with only three observations outside of the “agreement limits.” These three participants had no distinguishing characteristics. There was no obvious bias in the difference by the mean measurements. Thus, the two measurements of adherence were in excellent agreement and generally reflective of each other.

It is also interesting to note that the device has a built-in program to deliver 10 minutes of treatment or to “shut off” when a participant steps off the platform. When we examined the number of minutes per session using the electronic monitor data, we observed that participants generally remained on the platform for the entirety of the 10 minute session with only 0.5% of sessions ending prior to the 10 minutes. Twenty three percent of these occurrences can be attributed to one individual who routinely cut her sessions short.

## Discussion

In this study we compared two approaches to monitoring adherence to an intervention requiring daily attendance for a 10 minute session on a vibrating platform. One approach relied on self-reported use of the vibrating platforms via a sign-in logbook, and the other enlisted RFID card technology to create an electronic log. As new behavioral interventions become available for treatment, it is particularly relevant to know whether simple self-reporting using the equivalent of a log book may suffice compared to more sophisticated electronic monitoring approaches.

We found a very high correlation between electronic and self-reported adherence (ICC = 0.96). While electronic monitoring of device studies has not been previously described, electronic recordings of medication adherence using MEMS caps have been studied, often in comparison to calendars, journals, or questionnaires that involve recall of missed doses. Similar to previous medication-based studies [1,4,8,14-16], our self-reported adherence rate in this device trial was slightly higher than the electronic monitoring approach; however, the difference was minimal. Of note, self-generated reports of adherence in this study were created by participants signing a log book daily for each of their treatment sessions. As previously seen, this method would be expected to create more accurate reports of adherence than previous adherence studies that depended on participant’s retrospective recall of their session. [17]

Devices such as the platforms used in the VIBES trial are good candidates for electronic monitoring since they can be equipped with small, internally integrated memory components, although this may not be typical for other treatments. While we found excellent agreement between the two adherence monitoring approaches, admittedly, there may be good reasons to recommend using electronic monitoring as a preferred option to self-reporting when possible. First, electronic monitoring may reduce subject burden and the possibility for error as well as prevent inaccurate reporting. Furthermore the two participants who were excluded from comparison analyses due to personal characteristics that interfered with their ability to sign into the paper log sheet, highlights that in certain situations electronic monitoring can overcome physical characteristics that make the use of log books difficult. Further, device monitoring similar to that used in the VIBES trial may allow for a more complete picture of the treatment event, especially when accurate duration of treatment is desirable. In our case, electronic monitoring provided data regarding the actual time spent using the device, revealing one participant who had been initiating sessions regularly but not completing the entire 10 minutes specified by the protocol. The duration of treatment could be requested of self-reporters as well, albeit with less specificity than electronic recording provides. In spite of this, electronic monitoring may not always be feasible. While the reduced specificity in adherence tracking inherent in self-report is not ideal, our results suggest such monitoring to be sufficient in the tracking of behavioral routines.

While there are advantages to employing electronic monitoring, it does not come without limitations. On several occasions the equipment did not initiate a session when a participant attempted to start the machine via the RFID reader. This situation was typically due to the machine being unplugged, which could be easily fixed by resetting. Also, as described previously and observed in this study, electronic adherence records would have been inaccurate had we not solved the device’s clock malfunction (which required use of the log book self-reports). Again, this is a potential limitation of electronic device monitoring systems that would need to be rectified by improvements in the monitoring hardware and software. Another limitation to relying on electronic adherence data was that participants were required to use cards to activate the device; while few lost their card (n=12), participants occasionally used a card belonging to another individual assigned to the same machine, despite being asked not to do so. This situation occurred typically with spouses who had access to each other’s cards. These issues should be anticipated in future applications of this technology so that such practices are minimized. This may be feasible through alternative approaches such as body weight detection, cornea scans, or fingerprints, though the cost may be prohibitive. Due to these limitations, and based on the high concordance between use of a log book and an electronic monitoring approach, the use of a low cost, low tech log book may be preferred over more complex adherence monitors, especially in real-world practice settings.

## Conclusions

Behavioral interventions that involve a daily commitment are difficult to verify. Previous studies have often elicited the use of calendars or questionnaires to record adherence, but these have been shown to be less than reflective of actual adherence. We have shown that daily logs may be an accurate measure of adherence, as verified with electronically recorded adherence. Log books provide a simple, low cost, visual reference for participants and potentially serve as a motivational device as they display sessions completed and missed. Such reminder interventions can immediately and significantly improve adherence to treatment, even in individuals with high adherence rates [18]. In turn, this should reduce the complications often associated with poor adherence, and ultimately, reduce the cost of health care. While electronic monitors offer the advantage of being able to record events such as pauses or abbreviated sessions, and to overcome errors in self-reporting, low cost log books may be sufficient to monitor adherence. Though remote monitoring of devices can be expected with the advances in wireless technology and the internet, for lower tech interventions, log books can be implemented as an effective tool to track adherence to behavioral interventions.

## Competing interest

Dr. Kiel formerly served as an unpaid member of the Scientific Advisory Board for Marodyne Medical, LLC. Dr. Rubin is a founder of Marodyne Medical, LLC.

## Authors' contributions

BAJ substantially contributed to drafting the manuscript, contributed to the operations of the study, study data collection, interpretation of data, and led the preparation of the manuscript. MTH substantially contributed to the operations of the study, study design, and contributed to the preparation of the manuscript. EKQ contributed to statistical analyses, data analysis and contributed to the preparation of the manuscript. SZ substantially contributed to interpretation of the data and preparation of the manuscript. BAB contributed to the statistical analysis, interpretation of data, and preparation of the manuscript. CTR contributed in the conception, study design and preparation of the manuscript. DPK helped conceive and design the study, directed study operations, and contributed to the data analyses and preparation of the manuscript. All authors read and approved the final manuscript.

## Pre-publication history

The pre-publication history for this paper can be accessed here:

http://www.biomedcentral.com/1471-2288/12/171/prepub

## References

[B1] Farmer KC Methods for measuring and monitoring medication regimen adherence in clinical trials and clinical practice Clin Ther 1999 21 1074 90 discussion 1073. 10.1016/S0149-2918(99)80026-5 10440628

[B2] Osterberg L Blaschke T Adherence to medication N Engl J Med 2005 353 487 97 10.1056/NEJMra050100 16079372

[B3] Dunbar-Jacob J Mortimer-Stephens MK Treatment adherence in chronic disease J Clin Epidemiol 2001 54 Suppl 1 S57 60 11750211 10.1016/s0895-4356(01)00457-7

[B4] Wagner GJ Predictors of antiretroviral adherence as measured by self-report, electronic monitoring, and medication diaries AIDS Patient Care STDS 2002 16 599 608 10.1089/108729102761882134 12542933

[B5] Conn VS Interventions to improve medication adherence among older adults: meta-analysis of adherence outcomes among randomized controlled trials Gerontologist 2009 49 447 62 10.1093/geront/gnp037 19460887

[B6] McDonnell PJ Jacobs MR Hospital admissions resulting from preventable adverse drug reactions Ann Pharmacother 2002 36 1331 6 12196047 10.1345/aph.1A333

[B7] Martin S A comparison of adherence assessment methods utilized in the United States: perspectives of researchers, HIV-infected children, and their caregivers AIDS Patient Care STDS 2009 23 593 601 10.1089/apc.2009.0021 19591601 PMC2859776

[B8] Lui H A comparison study of multiple measures of adherence to HIV protease inhibitors Annals of Internal Medicine 2001 134 968 977 11352698 10.7326/0003-4819-134-10-200105150-00011

[B9] Rosen MI Improved adherence with contingency management AIDS Patient Care STDS 2007 21 30 40 10.1089/apc.2006.0028 17263651

[B10] Perno CF Virologic correlates of adherence to antiretroviral medications and therapeutic failure J Acquir Immune Defic Syndr 2002 31 Suppl 3 S118 22 12562033 10.1097/00126334-200212153-00006

[B11] Kiel DP Insights from the conduct of a device trial in older persons: low magnitude mechanical stimulation for musculoskeletal health Clin Trials 2010 7 354 67 10.1177/1740774510371014 20571129 PMC3136380

[B12] Blessed G Tomlinson BE Roth M The association between quantitative measures of dementia and of senile change in the cerebral grey matter of elderly subjects Br J Psychiatry 1968 114 797 811 10.1192/bjp.114.512.797 5662937

[B13] McGraw KO Wong SP Forming inferences about some intraclass correlation coefficients Psychological Methods 1996 1 30 46 Correction, Vol. 1, No. 4, 390.

[B14] Nakonezny PA A comparison of various methods of measuring antidepressant medication adherence among children and adolescents with major depressive disorder in a 12-week open trial of fluoxetine J Child Adolesc Psychopharmacol 2010 20 431 9 10.1089/cap.2009.0108 20973714 PMC3000641

[B15] Walsh JC Mandalia S Gazzard BG Responses to a 1 month self-report on adherence to antiretroviral therapy are consistent with electronic data and virological treatment outcome AIDS 2002 16 269 77 10.1097/00002030-200201250-00017 11807312

[B16] Arnstein J Antiretroviral therapy adherence and viral suppression in HIV-infected drug users: comparison of self-report and electronic monitoring HIV/AIDS 2001 33 1417 1423 10.1086/323201 PMC2692641 11550118

[B17] Garber MC The concordance of self-report with other measures of medication adherence: a summary of the literature Medical care 2004 42 649 10.1097/01.mlr.0000129496.05898.02 15213489

[B18] Sabin LL Using electronic drug monitor feedback to improve adherence to antiretroviral therapy among HIV-positive patients in China AIDS Behav 2010 14 580 9 10.1007/s10461-009-9615-1 19771504 PMC2865631

